# Prevalence of syphilis and associated factors among female sex workers in Ethiopia: findings from a multilevel analysis of a national bio-behavioral survey

**DOI:** 10.1186/s12889-023-15745-1

**Published:** 2023-05-03

**Authors:** Jaleta Bulti Tura, Jemal Ayalew, Ammar Barba Moreda, Sileshi Lulseged, Mohammed Ahmed Rameto, Lemessa Negeri Debel, Birra Bejiga Bedassa, Gemechu Gudeta Ebo, Feyiso Bati Wariso, Wudinesh Belete Belihu, Edosa Amente Gutema, Abebe Habteselassie, Getachew Tollera, Mesay Hailu, Saro Abdella Abrahim

**Affiliations:** 1 Ethiopian Public Health Institute, Addis Ababa, Ethiopia; 2 Wollo University, Addis Ababa, Ethiopia; 3 Faculty of Medicine, College of Health Sciences, Addis Ababa University, Addis Ababa, Ethiopia

## Abstract

**Background:**

Syphilis is a highly contagious sexually transmitted infection posing a significant public health challenge, especially in developing countries, including sub-Saharan Africa. Female sex workers are exposed to sexually transmitted infections, including syphilis, because of their sexual behavior and limited access to health services. However, data on national syphilis prevalence estimates and the associated factors are scarce in Ethiopia. This, as well as our limited knowledge about the extent of clustering among female sex workers in the country, is a critical gap in information we aimed to fill through this analysis.

**Methods:**

The study was a cross-sectional, bio-behavioral survey conducted among female sex workers in six cities and ten major towns in Ethiopia. Participants were selected using a respondent-driven sampling method. Survey participants provided blood samples for syphilis, HIV, and hepatitis serological testing. Survey data were collected via an interviewer-administered questionnaire. In this analysis, we employed descriptive statistics to summarize data on the study variables. In addition, we used multilevel bivariable and multivariable logistic regression models to examine the association between independent variables and the dependent variable (syphilis prevalence) while accounting for the clustering effect.

**Result:**

A total of 6085 female sex workers participated in the survey. Their median age [Interquartile Range (IQR) was 25 (8)] years, and a majority (96.1%) were in the 20–24-year-old age group. The prevalence of syphilis among female sex workers in Ethiopia’s six cities and ten major towns was 6.2%. Being in the age group of 30–34 (AOR = 2.64; 95% CI = 1.40, 4.98) and 35–59 (AOR = 4.7; 95% CI = 2.5, 8.86), being divorced/widowed (AOR = 1.37; 95% CI = 1.03, 1.82), having no formal education (AOR = 3.38; 95% CI = 2.34, 5.11), primary 1st cycle (grades 1–4) education (AOR = 2.77; 95% CI = 1.79, 4.30), and having primary 2nd cycle (grades 5–8) education (AOR = 1.80; 95% CI = 1.21, 2.69) were significantly associated with syphilis among female sex workers.

**Conclusion:**

The prevalence of syphilis among female sex workers was high. Being divorced/widowed or in the older age group and having a low level of education were significantly associated with an increased risk of syphilis. The high prevalence and associated factors identified need to be considered in planning comprehensive interventions to control syphilis among female sex workers in Ethiopia.

## Background

According to the World Health Organization’s (WHO) 2017 estimate, there were 18 million prevalent cases of syphilis worldwide [1]. Syphilis was a significant public health problem before the antibiotic era and remained so to date, particularly with the increase in the magnitude of acquired immunodeficiency virus. New knowledge has been generated regarding the high rates of syphilis and HIV co-infection [2, 3]. Syphilis spreads more easily in marginalized communities where mobility, violence, and alcohol abuse are prevalent, which is a bridge for unprotected sex [4–6]. The infection is transmitted through sexual activity, vertically from the mother to the fetus, via blood transfusion, and occasionally through direct contact with infectious lesions. Syphilis is one of the sexually transmitted infections (STI) caused by the bacterium *Treponema pallidum* that can cause acute illness, long-term complications, infertility, medical as well as psychological consequences, and death [7, 8]. The disease is also a significant cause of morbidity and mortality in pregnant women, especially in developing countries including Sub-Saharan Africa (SSA) [1, 4].

The sex sector is a leading contributor to syphilis and other STI, with 65% of HIV infections caused by key population (KP) groups around (sexual workers, injecting drug users, men who have sex with men, and transgender people [9]. Among the KPs, female sex workers (FSW) have a high STI rate than the general population and are more vulnerable to HIV, syphilis, and other STIs than the general population [10]. FSWs need special attention; because the disease affects their health [11], they can also spread STIs to their sexual partners, clients, children, and the general public [12]. FSW was 4.2% in Hawasa town, Southern Ethiopia, [13], and 12.5% in Dessie town, Northern Ethiopia [14]. Syphilis remains a significant cause of reproductive morbidity and poor pregnancy outcomes in low-income countries [1]. Stillbirth, perinatal death, neonatal severe infection, and low-birth-weight babies are common among syphilis seropositive mothers [1]. In a study on the public health impact of HIV and other STI in Ethiopia, the prevalence of syphilis among HIV-positive adults was 13.4% (17.4% among men and 11.5% among women), and of active syphilis was 2.6% (5.0% among men and 1.4% among women) [15]. The syphilis prevention and control strategy (2016–2021) set by the WHO aims to eliminate congenital syphilis by screening and treating pregnant women and specific populations such as those at risk and KP and priority populations (PP)- [16].

The epidemiology of syphilis indicates that the magnitude of syphilis has increased in selected high-income population groups, while the disease has remained endemic in low and middle-income countries [6]. The extent of syphilis in Nepal has remained at the same level [10] while increasing trends were observed in Eastern China from 3.19% to 4.47% [12]. A study in India reported a decline in syphilis prevalence from 5.9% to 2.4% [17]. A 2014 antenatal clinic (ANC)-based sentinel surveillance in Ethiopia showed a relative increase in syphilis prevalence among pregnant women from 1.0% in 2012 to 1.2% in 2014 [18].

The few studies conducted in Ethiopia were limited to single towns. Moreover, most of the studies in Ethiopia and other countries focused on individual-level factors or had limitations in accounting for intra‐cluster correlation, which falsely increases the precision of the estimates [19]. Therefore, this study aimed to assess the prevalence and critical determinants of syphilis while accounting for clustering effects. This approach provides evidence of the current prevalence and determinants of syphilis among FSW that will inform STI policies and preventive strategies.

## Materials and method

### Study design and setting

This analysis involved cross-sectional, bio-behavioral survey data collected using a respondent-driven sampling (RDS) technique, an approach to recruiting a hidden and difficult-to-reach population [20]. The survey was conducted in Ethiopia, from December 2019 to May 2020.

### Study participants

The participants were FSW aged ≥ 15 years who were residing in six regional capitals and 10 major towns in the month preceding the survey. The survey included fixed (venue-based) and floating (street-based) FSW.

### Sample size and sampling procedure

The study sample size was determined by single population proportion formula, Assuming a 95% confidence interval, α = 0.05, margin of error (d) = 35%, proportion (p) = 2% [15], and design effect of 1.5 with a replacement for non-responders. The minimum desired sample size of FSW in the six regional capitals and 10 major towns, and a 10% contingency, was 6085 FSW. This was allocated to the 16 sites proportionate to population size. Specific hotspot areas for FSW were identified during tools and procedures pretesting with support from HIV/AIDS Prevention and Control Office (HAPCO), woreda (district) health offices, and drop-in-clinics (DICs), local organizations working with FSW. We used a respondent-driven consecutive sampling using a standardized questionnaire to recruit study participants. The local organizations assisted in identifying the initial respondents of the survey referred to as “seeds”. The number of seeds for each site was determined based on the result of a formative assessment. Five "seeds" were recruited from each site with a sample of 450, six to eight seeds from each site with a sample of 450–900, and 12 seeds from each site with a sample of 1101. The “seeds” were selected based on the type of sex worker, age category, and geographic location of the site. These include those FSW who were bar- and/or hotel-based, red lighthouses, local drinking houses, street-based and hidden (cell phone-based). FSW, with a known social network, was given three coupons to invite other FSW contacts in her network.

Accordingly, among the invitees, those who fulfilled the following inclusion criteria were included in the study: had received money/other benefits for any type of sex with four or more people within the last 30 days, agreed to participate in the survey including questionnaire and biological testing, and age ≥ 15 years, was able to provide informed consent and resides or worked in the city for the last one month. This approach helped in reaching as many eligible FSW as possible. The coupon remained active from the day it was given to the potential participant and expired after two weeks or if the study was completed earlier. We used an anonymous fingerprint-based code obtained using biometric fingerprint scanners to ensure that all respondents participated only once. This unidentified fingerprint was not linked to the biobehavioral questionnaire and was used only to avoid multiple enrollments.

Damaged, mutilated, not readable, photocopied, and not sealed/stamped coupons were considered invalid. Each participant who comes to the study site would need to bring their coupon that was identified by a specific number given by the referring person. New participants were given vouchers and asked to recruit three additional FSW. This process continued until the desired sample size was achieved and the RDS equilibrium condition attained. There was monitoring progress toward equilibrium using key parameters, including current HIV status, type of sex work, and consistent condom use. The study sites, seeds, and sample size are presented in Table 1.

**Table 1:** Sample sizes and seeds distribution by study sites, national HIV and other sexually transmitted infections bio-behavioral survey, Ethiopia, 2019–2020 (*N* = 6085)

Study site	Sample size	Seeds	Study site	Sample size	Seeds
Addis Ababa	1101	13	Jimma	254	5
Adama	676	8	Arba Minch	251	5
Hawassa	522	8	Kombolcha/Dessie	251	5
Gambella	468	6	Dilla	251	5
Dire Dawa	434	5	Logia/Semera	251	5
Bahir Dar	372	8	Gonder	250	5
Nekemte	257	5	Shashemane	250	5
Mizan	255	5	Harar	242	5
Total				6085	98

### Data collection and procedure

For data collection, the content of the individual consent form and questionnaire was, initially developed in English and was translated into Amharic and Afan Oromo, the two most spoken languages in the country. First, all the survey teams were trained and assigned according to their roles and responsibilities (coordinators, interviewers, blood sample collectors, coupon managers for RDS, receptionists for RDS, and accompanying referral liaisons). Next the data was collected by trained data collectors using a pretested structured questionnaire. The data collectors read load each question for the study participants and recorded their responses on open data kits (ODK) software on tablet computers. Then supervisors monitored the overall data collection process after training on the survey's objective, methods, procedures, and ethical considerations.

### Testing for syphilis

We used Chembio Dual Path Platform, a rapid test kit, to detect Treponema Pallidum, and once the client was positive for Chembio Dual Path Platform rapid test kit, they were escorted to health facilities. At health facilities, the clients were re-tested for syphilis with the VDRL test. The client who tested positive for VDRL was diagnosed with active syphilis and received their results and was treated for syphilis infection.

### Data analysis

The data on the ODK software were exported to MS Excel, cleaned, and imported into STATA Verssion16 for analysis. R statistical software was used to implement the RDS recruitment process (Tree of recruitment), assessment of the RDS assumptions, and generate weights. Homophiles and convergence, the common assumptions in RDS, were checked for HIV status, consistent condom use, and type of FSW to ensure that they met the criteria. The RDS weights were exported using the RDS-II function to STATA and merged with the whole dataset for further analysis. STATA software calculated descriptive statistics like the crude and RDS-adjusted frequency, mean and standard deviations. Data collection took place from 16 different geographic areas, syphilis prevalence, and the associated behaviors would be affected by the unobserved site (city/town)-level factors and there was a high level of variation in health priorities and practices by region mainly because of the decentralized administration in Ethiopia. Therefore, models used for analysis had to account for associations among observations within clusters to make efficient and valid inferences. A fitted model was A for multilevel logistic regression to assess for variation by town and identify their association with the independent variables considered in the study.

In this analysis, a two-level multilevel mixed-effect logistic regression model was employed. All the independent variables categorized as individual-level variables were considered level-1 variables and the towns as level-2 variables. We used intra-class correlation (ICC) to quantify the effect of the level-2 variables (town) in the multilevel regression model, and the between-town variation accounted for the proportion of total variation in the response variable. The effects of individual-level predictors were quantified by the estimates from the mixed-effect part of the model with a *P*-value less than 0.05 or 95% CI that didn’t include unity.

### Ethical approval and consent to participate

The survey and all experimental protocols were reviewed and obtained ethical clearance from the Scientific and Ethical Research Office (SERO) of the Ethiopian Public Health Institute (EPHI) with protocol number EPHI-IRB-108–2018 on meeting no.27 and approved on the date 01 September 2018. In addition, guidelines and regulations of research ethics and principles of the Declaration of Helsinki governed all methods used.

Before performing study-specific procedures, we obtained consent from each participant using local languages, and volunteers opted to consent in the language of their preference. Copies of the signed informed consent were given to the participants for their records for sample storage before participation.

## Results

A total of 6085 FSW participated in the survey. Their median age [Interquartile Range (IQR) was 25 [8] years, and majorities were in the 20–24 years age group and attended primarily the 2^nd^ cycle (grade 5–8). Table 2.

**Table 2:** Demographic and behavioral characteristics of female sex workers, national HIV and other sexual transmitted infections bio-behavioral survey, Ethiopia, 2019 -2020 (*N* = 6085)

Variables		Frequency	Percent
Age (years), Median (IQR)	25 (8)		
Age in Year	15 – 19	615	10.1
	20 – 24	1980	32.5
	25 – 29	1815	29.8
	30 – 34	856	14.1
	35 – 59	819	13.5
Level of education	Non-formal Education	1054	17.3
	Primary 1st cycle (grade 1–4)	848	13.9
	Primary 2nd cycle (grade 5–8)	2712	44.6
	Secondary school and above	1471	24.2
Marital status	Married/Cohabitation	231	3.8
	Divorced/Separated/Widowed	2908	47.8
	Never married	2946	48.4
Ever been pregnant	No	1873	30.8
	Yes	4212	69.2
Number of pregnancies	0	1873	30.8
	1	2059	33.8
	2	1238	20.3
	3 +	915	15
Ever been pregnant	No	1873	30.8
	Yes	4212	69.2
Number of pregnancies	0	1873	30.8
	1	2059	33.8
	2	1238	20.3
	3 +	915	15
Currently pregnant	No	5973	98.2
	Yes	112	1.8
The main source of income	Other than sex work	391	6.4
	Sex work	5694	93.6
Average monthly income from selling sex (ETB)	< 2500	1778	29.2
	2500 – 4999	2066	34
	5000 – 7499	1175	19.3
	7500 +	1066	17.5
Age at first sexual intercourse (years), median (IQR)	16 (3)		
Age at first sexual intercourse (years), n (%)	≤ 15	2430	39.9
	16–20	3384	55.6
	≥ 21	271	4.5
Age at first sex selling	< 20	2328	38.3
	20–24	2348	38.6
	≥ 25	1406	23.1
Age of first sex partner	5 or more years younger	73	1
	About the same age	2335	38.4
	5–10 years older	2425	39.9
	More than 10 years older	1252	20.6
First sex experience	Wanted	4738	77.9
	Forced	1347	22.1
Number of paying partners in the last 6 months	≤ 30	2308	37.9
	31 – 60	1436	23.6
	61 – 90	696	11.4
	≥ 91	1645	27
Ever had Anal intercourse	Yes	426	7
	Never	5659	93
Used condom during anal sex	Yes	252	59.2
	No	174	40.8
Consistent Condom utilization during the last 30 days with paying clients	Yes	5119	84.1
	No	966	15.9
HBV among non-condom users during the last 30 days with paying clients	HBsAg positive	23	2.4
	HBsAg negative	943	97.6
HBV among condom users during the last 30 days with paying clients	HBsAg positive	134	2.7
	HBsAg negative	4651	97.3
Breakage of condom during the last 30 days	No breakage	4260	70
	Experienced breakage	1825	30
In the last 30 days, used cigarettes or cigars	No	5343	87.8
	Yes	742	12.2
Alcohol consumption level (AUDIT scores)	Not Risky	2594	42.8
	Harmful, hazardous drinking	1210	20
	Alcohol dependence indication	2257	37.2
Chewing khat in the last 30 days	Yes	3827	62.9
	No	2258	37.1
Any drug used other than alcohol and khat in the last 30 days	Yes	700	11.5
	No	5382	88.4
Used shisha in the last 30 days	Yes	856	14.1
	No	5229	85.9
Depression level	Not depressed	2468	40.6
	Mild depression	2525	41.5
	Moderate to severe depression	1092	17.9
HIV test result	Tested negative	4945	81.3
	Tested new positive	565	9.3
	Already known positive	575	9.4

### Prevalence of syphilis

A high proportion of syphilis infections (95.5%) were observed among FSW who were not living with their sexual partner (Table 3). The highest proportion (35.7%) of FSW who were positive for syphilis was between the ages of 35 and 59. The highest proportion of syphilis (69.6%) was among divorced/separated/widowed, followed by 26.3% among never married FSW. Among FSW who tested positive for syphilis, the highest proportion 54.6% were in primary education followed by 53.4% of FSW with non-formal education. The proportion of syphilis infections decreased as the average monthly income from selling sex increased.

**Table 3:** Socio-demographic characteristics of female sex workers testing positive for syphilis infection, national HIV and other sexual transmitted infections bio-behavioral survey, Ethiopia, 2019–2020 (*N* = 6085)

Characteristics	Non-reactive	Reactive for syphilis		
	**Number**	**Number (%)**	*p*-value	95% CI
**Age categories**				
15—19	601	14 (4.1)	< 0.001	2.4, 6.7
20—24	1926	54 (15.9)		12.4, 20.2
25—29	1736	79 (23.3)		19.1, 28.1
30—34	785	71 (20.9)		16.7, 25.3
35—59	698	121 (35.7)		30.8, 41.0
**Marital status**				
Married/Cohabitation	217	14 (4.1)	< 0.001	2.4, 6.7
Divorced/Separated/Widowed	2672	236 (69.6)		64.5, 74.3
Never married	2857	89 (26.3)		21.9, 31.2
**Highest educational status attained**				
Non-formal Education	934	120 (35.4)	< 0.001	30.5, 40.7
Primary school (grade 1–8)	3375	185 (54.6)		49.4, 60.0
Secondary school and above	1437	34 (10.0)		6.9, 13.3
**Living with sexual partner**				
No	5263	324 (95.6)	0.009	93, 97.4
Yes	483	15 (4.4)		2.6, 7.0
**Main source of income**				
other than sex work	357	34 (10.0)	0.005	7.2, 13.6
Sex work	5389	305 (90.0)		86.4, 92.8
**Average monthly income from selling sex in ETB**				
< 2500	1619	159 (46.9)	< 0.001	41.5, 52.1
2500—4999	1950	116 (34.2)		29.4, 39.5
5000—7499	1138	37 (11.0)		8.0, 14.6
7500 +	1039	27 (7.8)		5.5, 11.2

### Prevalence of syphilis in cities and major towns

The prevalence of syphilis among FSW in six cities and 10 major towns was 6.2% (Table 4). The majority was in Dilla (14.1%), Shashimane (11.5%), and Jimma (10.6%), and low in Addis Ababa (3.9%), Harar (3.7), Adama (3.3%), and Gambella (2.5%), while it ranged from 4.5% to 8.8 for the other cities and major towns.

**Table 4:** Prevalence of active syphilis among female sex workers, national HIV, and other sexually transmitted infections bio-behavioral survey, Ethiopia, 2019–2020 (*N* = 6085)

**Study site**	**Participants**			
	**Number tested**	**Number reactive**	**Percent reactive**	**95% CI for reactive**
Dilla	251	31	14.1	11.8, 16.9
Shashemane	250	27	11.5	9.3, 14.0
Jimma	254	25	10.6	8.5, 12.7
Mizan	255	21	8.8	7.0, 11.1
Arba Minch	251	26	8.4	6.5, 10.7
Hawassa	522	43	6.7	5.1, 8.8
Nekemite	257	10	6.5	4.1, 9.9
Logia/Semera	251	15	6.3	4.6, 8.3
Combolcha/Dessie	251	11	5.8	3.4, 9.0
Diredawa	434	20	5.0	3.8, 6.6
Bahir Dar	372	17	4.8	3.1, 7.0
Gonder	250	10	4.5	2.9, 6.6
Addis Ababa	1,101	41	3.9	3.1, 4.6
Harar	242	11	3.7	2.5, 5.3
Adama	676	21	3.3	2.5, 4.3
Gamebella	468	10	2.5	1.4, 4.2
Total	6085	339	6.2	5.8, 6.6

### Sexual history

The FSW who described their first sex experience as forced had the highest syphilis positivity (7.9%). FSW with known and new HIV-positive status had high syphilis positivity at 19.2% and 12.9% respectively (Table 5). Prevalence of syphilis positivity status was high (8.1%) among those with a history of sexual intercourse without a condom, in the last 1 month and 7.8% among clients who experienced condom failure in the past one month.

**Table 5:** Syphilis prevalence by sexual history and behavior among female sex workers, national HIV and other sexual transmitted infections bio-behavioral survey, Ethiopia, 2019–2020 (*N* = 6085)

**Study variable**	**Non-reactive**	**Reactive**	95% CI
	Number	No. (%)	
**First sex experience**			
Wanted	4498	240 (5.3)	4.9, 5.8
Forced	1248	99 (7.9)	8.2, 10.4
**Number of sexual partners without condoms in the last 30 days**			
Have sex without a condom	894	72 (8.1)	6.6, 8.8
Always used condom	4852	267 (5.5)	5.5, 6.3
**Experienced condom failure in the last 30 days**			
No	4053	207 (5.1)	4.7, 5.6
Yes	1693	132 (7.8)	7.9, 9.7
**Number of non-paying partners in the past 6 months**			
Never	4106	241 (5.9)	5.5, 6.4
Only one	1324	80 (6.0)	5.6, 7.4
2 and more	316	18 (5.7)	6.4, 10.1
**Used lubricant during sex in the last 6 months**			
Not use	5014	286 (5.7)	5.5, 6.4
Yes used	732	53 (7.2)	6.5, 9.1
**Given lubricants free of charges**			
Not given	5318	314 (5.9)	5.9, 6.7
given	428	25 (5.8)	3.6, 6.3
**At least two STI symptoms occurred in the last 12 months**			
No	4809	274 (5.7)	5.6, 6.5
Yes	937	65 (6.9)	5.9, 8.1
**Ever been raped or forced to have sex in the past 12 months**			
No	5022	292 (6.1)	5.7, 6.5
Yes	724	47 (6.5)	5.8, 8.2
**HIV comprehensive knowledge**			
No	5022	292 (5.8)	6.0, 7.0
Yes	724	47 (6.5)	4.5, 6.0
**Ever tested and received HIV test result**			
Ever tested and received test result	5146	300 (5.8)	5.6, 6.4
Never tested or received test result	600	39 (6.5)	6.5, 9.2
**Age at first sex selling**			
< 20	2243	85 (3.8)	4.1, 5.3
20—24	2229	119 (5.3)	4.8, 6.1
25 +	1272	134 (10.5)	8.7, 10.8
**Age at first sex**			
15 or less	2265	165 (7.3)	6.8, 8.2
16—20	3223	161 (5.0)	4.9, 5.9
21 +	258	13 (5.0)	4.1, 7.5
**Only street**			
No	3982	232 (5.8)	5.5, 6.5
**Yes**	1764	107 (6.1)	5.9, 7.4
**Only the bar and hotel**			
No	3798	264 (7.2)	6.6, 7.7
Yes	1948	75 (4.3)	3.7, 4.9
**Only Own home**			
**No**	5434	303 (6)	5.6, 6.5
**Yes**	312	36 (10)	7.7, 12.8
**HIV Status**			
Negative	4767	178 (4)	3.6, 4.4
Positive	979	161 (16)	14.6, 17.6
**Hepatitis B**			
Non-reactive	5602	326 (6.1)	5.7, 6.6
Reactive	144	13 (8.3)	5.7, 11.7
**Hepatitis C**			
**Non-reactive**	5726	332 (6)	5.6, 6.4
**Reactive**	20	7 (39.8)	28.7, 51.6
**Syphilis**			
**Non-reactive**	5746	0 (0)	
**Reactive**	0 (0)	339 (100)	
**HIV test result**			
Tested negative	4767	178 (4)	3.6, 4.4
Tested new positive	487	78 (19.2)	17.1, 21.6
Known positive	492	83 (12.9)	11.2, 15
**Highest educational status attained**			
Non-formal education	934	120 (13.2)	11.9, 14.6
Primary school (grades 1–8)	3375	185 (5.5)	5.0, 6.1
Secondary school and above	1437	34 (2.3)	1.8, 2.8
**Year lived as female sex workers**			
< 5 years	2215	93 (4.7)	4.2, 5.3
5—10 years	1458	101 (7.1)	6.2, 8.0
11 + yYears	2073	145 (7.2)	6.4, 7.9

### Socio-demographic and other associated factors

The results of the multilevel mixed-effect logistic regression model show that the model fitted well for the data over the standard logistic regression to assess determinants for syphilis prevalence ($P<0.001$). The result of the ICC for the two-level multilevel model revealed the variation explained about 9.6% of the likelihood of syphilis prevalence among the towns in Ethiopia. As we can see from the 95% CI (0.04, 0.20), the variation among the cities and major town towns is statistically significant, hence any estimation without considering this effect will result in a biased estimate (Table 6).

**Table 6:** COR* and AOR** from the multilevel mixed-effect logistic regression of syphilis and independent factors among female sex workers, national HIV and STI^£^ bio-behavioral survey, Ethiopia, 2019–2020

Characteristic	COR	*P*-value	AOR (95% CI)		*P*-value
**Age category**					
5 – 19	1		1		
20 – 24	1.37	0.307	1.34 (0.72, 2.46)		0.353
25 – 29	2.42	0.003	1.62 (0.88, 2.98)		0.120
30 – 34	5.2	< 0.001	2.64 (1.40, 4.98)		0.003
35 – 59	11.09	< 0.001	4.70 (2.50, 8.86)		< 0.001
**Marital status**					
Married/Cohabiting	2.07	0.016	1.70 (0.92, 3.13)		0.091
Divorced/Separated/Widowed	2.95	< 0.001	1.37 (1.03, 1.82)		0.033
Never married	1		1		
**Highest grade attended**					
Non-formal Education	5.69	< 0.001	3.38 (2.34, 5.11)		< 0.001
Primary 1^st^ cycle (grades 1–4)	3.6	< 0.001	2.77 (1.79, 4.30)		< 0.001
Primary 2^nd^ cycle (grades 5–8)	1.88	0.001	1.80 (1.21, 2.69)		0.004
Secondary school and above	1		1		
**Living with a sexual partner**					
No	1				
Yes	0.54	0.022			
**The main source of income**					
Other than sex work	1				
Sex work	0.66	0.036			
**Average monthly income from selling sex in ETB**					
< 2500	3.64	< 0.001	1.74 (1.09, 2.78)		0.020
2500—4999	2.26	< 0.001	1.41 (0.89, 2.23)		0.141
5000—7499	1.27	0.351	1.03 (0.61, 1.73)		0.920
7500 +	1		1		
**Alcohol consumption levels**					
Not depressed	1				
Mild Depression	0.97	0.83			
Moderate to severe depression	1.57	0.003			
**First sex experience**					
Wanted	1.43	0.003			
Forced	1				
**Number of cities worked sex selling in the last three years**					
Same town	1				
1 more town	0.68	0.036			
2 or more towns	0.65	0.088			
**Number of clients in the past 6 months**					
< 30	1.89	< 0.001			
31—60	1.51.252	0.233			
61—90	1.14	0.592			
91 +	1				
**Age at first sex selling**					
< 20	1				
20—24	1.55	0.003			
25 +	3.27	< 0.001			
**Age at first sex**					
15 or less	1.48	0.001			
16—20	1				
21 +	1.04	0.896			
**Only Bar or Hotel FSW**					
No	2.18	< 0.001	1.53 (1.45, 2.05)		0.004
Yes	1				
**Only Own home FSW**					
No	1				
Yes	3.04	< 0.001			
**Hepatitis B**					
Non-Reactive	1				
Reactive	1.58	< 0.001			
**Number of sexual partners without condoms in the last 30 days**					
Consistent	1				
In-consistent	1.38	0.028			
**Experienced condom failure in the last 30 days**					
No breakage	1				
Breakage	1.52	< 0.001			
**HIV test result**					
Tested Negative	1		1		
Tested New Positive	4.58	< 0.001	2.61 (1.92, 3.56)		< 0.001
Known Positive	4.92	< 0.001	2.41 (1.76, 3.3)		< 0.001
**Ever been pregnant**					
No	1				
Yes	2.78	< 0.001			
**Number of pregnancies**					
0	1				
1	2.13	< 0.001			
2	2.92	< 0.001			
3 +	4.56	< 0.001			
**Currently pregnant**					
No	1				
Yes	0.46	0.195			
**History of miscarriage pregnancy**					
No	1				
Yes	1.32	0.100			
**Constant**			0.004 (0.002, 0.01)		< 0.001
**Random effects**					
Towns/cities			0.35	0.15	0.80
ICC			0.096	0.044	0.20
**LR test vs. logistic model**					
chibar2 = 61.32					
*P*-value = < 0.001					

^*^Crude Odds Ratio ** Adjusted Odds Ratio £ Sexually Transmitted Infections

FSW aged 30–34 (AOR = 2.64; 95% CI = 1.40, 4.98) and 35–39 years (AOR = 4.7; 95% CI = 2.5, 8.86) were associated with syphilis than those in the age group 15–19 years, respectively. Being a divorced/widowed FSW (AOR = 1.37; 95% CI = 1.03, 1.82) was associated with syphilis compared to married/cohabiting. FSW with no formal education (AOR = 3.38; 95% CI = 2.34, 5.11), had primary 1^st^ cycle (grade 1–4) (AOR = 2.77; 95% CI = 1.79, 4.30), and had primary 2^nd^ cycle (grade 5–8), (AOR = 1.80; 95% CI = 1.21, 2.69) were associated with syphilis compared to those who had secondary school education and above. Regarding monthly income from sex work, an earning less than 2500 birr per month (AOR = 1.74; 95% CI = 1.09, 2.78) was significantly associated with syphilis compared to earning 7500 Birr or more. FSW who worked at a venue other than the bar or hotel was associated with being infected with syphilis compared to those who worked only at the bar or hotel. FSWs that tested positive for HIV for the first time or already knew their HIV status had an association with acquiring syphilis compared to those who tested negative for HIV.

## Discussion

In this survey, in which we assessed syphilis prevalence and associated factors among FSW, syphilis prevalence was high. It was associated age group of 30–34 and 35–59 divorced/widowed, having no formal education, being in primary 1^st^ cycle (grades 1–4) and primary 2^nd^ cycle (grades 5–8), and working at a venue other than the bar or hotel and being HIV positive.

When comparing the finding of this survey with other studies the finding result is similar to the prevalence reported from different sub-Saharan African countries like Burkina Faso (5.6%) [11] but higher than the figures reported from Nepal (3.9%), [10], Eastern China (4.31%) [12], and Somaliland (3.1%) [21]. On the other hand, the prevalence we identified is much lower than what has been reported from similar developing settings like Pará, Brazilian Amazon (14.1%) [22], and Rwanda (51.1%) [23]. Overall, published reports show that the prevalence of- syphilis varies across and within countries and this might be explained partly by the methods used in the different studies but might also be the effect of various risk factors in multiple settings.

There still is a scarcity of data from well-planned epidemiological studies on the prevalence and risks associated with the disease in the general population as well as high-risk groups like FSW in Ethiopia [24]. The Ethiopian population-based impact assessment (EPHIA) conducted in urban Ethiopia during 2017–2018 revealed syphilis prevalence among HIV-positive patients was 13.4% (17.4% in men and 11.5% in women) [15]. Similarly high prevalence among FSW has been reported in the Para State, Brazil Amazon [22], and Rwanda (51.1%) [23]. Even though not identified in our study, the possible reason for high syphilis among risk population groups could be inconsistent condom use [10], sexual violence, and power imbalance in condom use negotiations according to different studies [23, 25].

Our multilevel model revealed that factors like the age of FSW, marital status, average monthly income from selling sex, educational level of FSW, place of sex selling or workplace, and HIV infection status of FSW were significantly associated with syphilis in Ethiopia. In our study, as the age of the FSW increased the risk of acquiring syphilis also increased. The result of our research is in line with the finding of the study conducted in Nepal [10], which showed a strong association between the age of FSW and the risk of infection with Syphilis. Similarly, another study conducted in Dessie town, Northern Ethiopia, indicates the risk of syphilis infections was high among older FSW [14]. Even though older age and syphilis show associations, a focused study must discern the underlying reasons.

Our study also reveals that FSW with divorced/widowed/separate marital statuses are more likely to be infected with syphilis. A separate study found that married FSW were less likely to contract syphilis [22]. In contrast, a study found that being married or living with a partner increases the risk of syphilis [23]. This disparity could be because of social factors and or other cultural influences determining the acquisition of syphilis infection, but this requires further studies.

Low education level was a risk factor for syphilis among the FSW in our study. Similarly, studies in different African countries including Rwanda and Burkina Faso [11, 23] show that FSW with low educational levels were more likely to be infected with syphilis. In addition, the reports from Eastern China and India [9, 10], also show that the prevalence of syphilis is high among more disadvantaged FSW, as they had a significant number of clients and less frequent condom use. As emphasized by others [12, 23], this could be because FSW who attended secondary education or more could be more aware of syphilis prevention measures and use of health facilities for treatment and could be better positioned to negotiate on condom use, and might have, as suggested by our study, less exposure to the infection because they had an additional source of income.

In our study, FSW with less income under 2500 ETB were more likely to be infected with syphilis than those earning more than 2500 ETB from selling sex. This concurs with observations in other studies which showed that some economic characteristics, including income, were predictors of vulnerability to syphilis and other STI [26]. As suggested by another study [7], HIV co-infection is another factor associated with the increased prevalence of syphilis infection. HIV-positive FSW (newly identified HIV-positive and those with known HIV-positive status) are more likely to be infected by syphilis. This finding corroborates the research finding that syphilis is a biological risk factor for HIV infection [23]. Similarly, other studies also identified that syphilis is strongly associated with HIV infection [7, 15, 23]. Therefore, the primary explanation for the association between syphilis and HIV could be that both have a similar transmission mode.

Our finding also revealed that syphilis is higher among FSW who are working in venues other than bars or hotels, such as streets, red-light, and other places. The findings are consistent with those in the Nepali report, which indicates the FSW who work on the roads are more likely to contract syphilis [10]. In Ethiopia, working as FSW is considered illegal and unacceptable by the community, which results in FSW hiding themselves and poor condom use negotiation. In addition, a high proportion of street FSW had a low level of education which exposes them to the risk of syphilis and other STI infections.

### Limitations

The survey targeted only provincial (regional) capital cities and major towns as part of the ongoing surveillance. However, even in the selected cities, the presence of harder-to-reach sex worker groups like home-based sex workers might not be fully accounted for. As VDRL test is not always accurate, there is a possibility that some infections can trigger a false-positive result. Therefore, this needs to be taken into consideration in the interpretation of results related to this.

## Conclusion

Syphilis is prevalent among FSW in Ethiopia, though the study may not have reached all its targets. Factors like marital status, older age, and low educational background of the FSW were significantly associated with the risk of syphilis infection. The high prevalence and identified risk factors need a targeted and comprehensive intervention against syphilis/STI and strengthen the existing system including condom distribution and use in the community, linking FSW in the community to link them to care systematically and treatment services, and organizing income-generating activities to prevent and control syphilis among FSW in Ethiopia.

## Data Availability

Study data is available from the Ethiopian Public Health Institute (EPHI) upon request at the following address. Ethiopian Public Health Institute. Swaziland street (The former Arbegnoch street), Wereda 09, house number 626, Gulele sub-city, Addis Ababa. P. O. Box 1242/5654.
